# Evolutionary Diversity of Prophage DNA in *Klebsiella pneumoniae* Chromosomes

**DOI:** 10.3389/fmicb.2019.02840

**Published:** 2019-12-06

**Authors:** Fengling Wang, Dongguo Wang, Wei Hou, Qian Jin, Jiao Feng, Dongsheng Zhou

**Affiliations:** ^1^Department of Infectious Disease, Taizhou Municipal Hospital, Taizhou University, Taizhou, China; ^2^Department of Clinical Laboratory Medicine, Taizhou Municipal Hospital, Taizhou University, Taizhou, China; ^3^Institute of Biomedical Sciences, Shanxi University, Taiyuan, China; ^4^State Key Laboratory of Pathogens and Biosecurity, Beijing Institute of Microbiology and Epidemiology, Beijing, China

**Keywords:** *K. pneumoniae* chromosome, DNA conjugative transfer, sequence annotation and comparison, massive genetic acquisition or loss, evolutionary diversity of prophage DNA

## Abstract

Mobile gene elements play an important role in the continuous evolution of the prophage DNA of bacteria, promoting the emergence of new gene structures. This study explored the evolution of four strains of *Klebsiella pneumoniae* harboring prophages, 19051, 721005, 911021, and 675920, and 16 genomes of *K. pneumoniae* from GenBank. The results revealed a wide range of genetic variation in the prophage DNA inserted into the *sap* sites of *K. pneumoniae* chromosomes. From analysis and comparison of the sequences of the 20 prophage DNAs determined from the four strains and the 16 GenBank genomes of *K. pneumoniae* using high-throughput sequencing and antimicrobial susceptibility tests, we identified a novel transposon, Tn*6556*. We also identified at least nine large genetic structures with massive genetic acquisitions or losses and five hotspot sites showing a tendency to undergo insertion of gene elements such as IS*1T*, IS*1R*, IS*26*, IS*Kpn26*, IS*Kpn28*, Tn*6556*, MDR, and In27*-*related regions as variable regions; however, the only highly conserved core genes were *int* and *umuCD* among the 20 prophage DNAs. These findings provide important insights into the evolutionary diversity of bacteriophage DNA contained in *K. pneumoniae*.

## Introduction

Antimicrobial resistance (AMR) genes originate from environmental bacteria, especially from soil bacteria, which are thought to have evolved together with related antibiotic production organisms over thousands of years (Dcosta et al., 2011; Nesme et al., 2014). It usually takes several years of clinical use of drugs associated with mobile AMR genes for the genes to penetrate human pathogen populations (Lewis, 2013). Hundreds of mobile AMR genes have been found in *Klebsiella pneumoniae* (Holt et al., 2015; Navon-Venezia et al., 2017). The accumulation of AMR in these bacteria is mainly related to horizontal gene transfer assisted by plasmids and mobile gene elements (Pendleton et al., 2013), such as insertions (IS), transposons (Tn), and integrons (In). Evolutionary forces drive the growth of bacteria in many circumstances, and survival under less than optimal conditions is essential. Therefore, bacteria often acquire the ability to survive in the presence of an antimicrobial agent in an effort to adapt to their environment (Alanis, 2005).

Bacteriophages (phages) are viruses that infect bacteria; they constitute a large and diverse group within microorganisms. It is estimated that approximately 20% of the total bacterial genome has been obtained from phage elements, indicating an evolutionary correlation between bacteria and phages (Brüssow et al., 2004). Phages induce antiviral immunity and prevent the elimination of bacterial infections (Sweere et al., 2019). A number of studies have shown that in mammals, phages are also related to bacterial colonization (Davies et al., 2016; Shen et al., 2019).

Phages that integrate into bacterial chromosomes are called prophages, which are important gene elements of bacterial chromosomes and enable horizontal gene transfer between bacteria and phages (Bushman, 2002). The interaction between phages and bacteria may have evolutionary benefits because many bacterial species have phage-derived factors in their genomes that benefit their survival, and such genetic exchange can be promoted given the appropriate ecological conditions (Calero-Cáceres et al., 2019). For example, Wang et al. (2010) has described how prophage DNA can help the host survive under multiple adverse conditions including the presence of antibiotics and environmental stress. Prophages also encode the virulence factors of many pathogens (Castillo et al., 2018; Deutsch et al., 2018). Attempts have been made to use phages to treat multidrug-resistant bacterial infections (Furfaro et al., 2018; Torres-Barceló, 2018; Dedrick et al., 2019; Kortright et al., 2019), but their function and significance remain unclear (Keen and Dantas, 2018). Only recently have studies confirmed that phages play more crucial roles than previously expected in the maintenance and dissemination of acquired resistance genes (Calero-Cáceres et al., 2019).

*Klebsiella pneumoniae* is an important conditional pathogen in hospitals and the environment. The global multidrug resistance (MDR) problem of *K. pneumoniae* is becoming more and more serious, and the antimicrobial treatment options for infection are limited (Rice, 2010). The role of prophages in highly virulent and multidrug resistant pathogens, including *K. pneumoniae*, has received much attention (Brown-Jaque et al., 2015). Building on previous studies, herein, four strains of *K. pneumoniae*, 19051, 675920, 721005, and 911021, from hospital patient specimens and 16 genomes of *K. pneumoniae* from GenBank were compared and analyzed for the presence of prophage DNA at the *sap* sites in an effort to confirm its importance as the basis of the horizontal transfer of resistance genes in the prophage DNA. A novel transposon, Tn*6556*, was discovered and the related properties of the prophage genes inserted into the *sap* sites of *K. pneumoniae* are described. This study describes the diversity of prophage genetic mutations, and deciphers the detailed molecular mechanisms of AMR for *K. pneumoniae* harboring prophage DNA.

## Materials and Methods

### Ethics Statement

This study was approved by the Ethics Committee of the Taizhou Municipal Hospital, Taizhou University, Zhejiang, China, and written informed consent was obtained from each of the participants in accordance with the Declaration of Helsinki. The rights of the research subjects were protected throughout, and we confirm that this study was conducted in our hospital.

The use of human specimens and all related experimental protocols were approved by the Committee on Human Research of the indicated institutions, and the protocols were carried out in accordance with approved guidelines.

### Bacterial Strains and Sequencing of the 16S rRNA Gene

*Klebsiella pneumoniae* strains 19051 and 721005 were isolated from urine samples of two patients in hospital in 2011 and 2013. *K. pneumoniae* strain 911021 was obtained from the urine of a patient in a teaching hospital in 2014. *K. pneumoniae* strain 675920 was obtained from a patient’s sputum in another hospital in 2015. *Escherichia coli* TOP10 (Invitrogen, Carlsbad, CA, United States) was used as the host for cloning and azide-resistant *E. coli* J53 was used as the recipient strain for the conjugation experiments.

For PCR amplification of the almost complete 16S rRNA genes of the *K. pneumoniae* strains, the following universal eubacterial primers were used: AGAGTTTGATYMTGGCTCAG (forward), and TACCTTGTTACGACTT (Y, T or C; M, A or C) (reverse). The length of the amplicon was about 1,500 bp (Frank et al., 2008). The Taq enzyme was a Fermentas Taq:Pfu 3:1 mixture (ThermoFisher Scientific, Burlington, VT, United States). The 30 μl reaction contained 1.5 U of enzyme. Amplification was carried out using a temperature program consisting of initial denaturation at 94°C for 3 min, 30 cycles of denaturation at 94°C for 40 s, annealing at 50°C for 40 s, extension at 72°C for 1 min, and final extension at 72°C for 5 min. The PCR products were bidirectionally sequenced to confirm their identity.

### Conjugative Transfer of Prophage DNA

Sodium azide-resistant *E. coli* J53 was used as a receptor, and the prophage-containing *K. pneumoniae* strains 19051, 675920, 721005, and 911021 were used as donors for the conjugative transfer experiments. Small amounts (10–20 μl) of donor and recipient glycerol bacteria were inoculated in 3 ml of brain heart infusion (BHI) broth (BD Biosciences, San Jose, CA, United States). After culture overnight, the donor bacteria were mixed with the recipient bacteria, centrifuged, resuspended in 80 μl of BHI broth, supplemented with a resuspended bacterial solution at the surface of a filter (filter size 1 cm^2^, pore size 0.45 μm), and the filter was adhered to BHI (BD Biosciences) agar plates. The cells were fully absorbed by the filter and cultured at 37°C for 12–18 h. The bacteria were then washed from the filter and spotted on to the BHI agar plates containing 200 μg/ml sodium azide and 50 μg/ml amikacin to select conjugons harboring the prophage.

### Sequencing and Sequence Assembly

Genomic DNA was isolated from each of the 19051, 721005, and 911021 isolates using a Qiagen Blood & Cell Culture DNA Maxi Kit (Qiagen, Hilden, Germany). Genome sequencing was performed with a sheared DNA library with an average size of 15 kb (ranging from 10 to 20 kb) on a PacBio RSII sequencer (Pacific Biosciences, Menlo Park, CA, United States), as well as a paired-end library with an average insert size of 400 bp (ranging from 150 to 600 kb) on a HiSeq sequencer (Illumina, San Diego, CA, United States). The paired-end short Illumina reads were used to correct the long PacBio reads utilizing proovread (Hackl et al., 2014), and then the corrected PacBio reads were assembled *de novo* utilizing SMART *de novo*^1^.

For the 675920 isolate, genome sequencing was performed with a Qiagen large construct kit with sequencing from a mate-pair library with average insert size of 5,000 bp using a MiSeq sequencer (Illumina, San Diego, CA, United States). Sequence assembly was performed as described previously (Feng et al., 2016). Briefly, the contigs were assembled using Newbler 2.6 (Nederbragt, 2014).

### Sequence Annotation and Comparison

Open reading frames and pseudogenes were predicted using *RAST*2.0 (Brettin et al., 2015), *BLASTP/BLASTN* (Boratyn et al., 2013), *UniProtKB/Swiss-Prot* (Boutet et al., 2016), and *RefSeq* databases (O’Leary et al., 2016). Annotation of drug resistance genes, mobile elements, and other features was performed using online databases such as *CARD* (Liang et al., 2017), *ResFinder* (Zankari et al., 2012), *ISfinder* (Siguier et al., 2006), *INTEGRALL* (Moura et al., 2009), and the Tn Number Registry (Roberts et al., 2008). Multiple and pairwise sequence comparisons were performed using *MUSCLE* 3.8.31 (Edgar, 2004) and *BLASTN*. The genome map was drawn using *Inkscape* 0.48.1^2^.

### Phenotypic Assays

The method used for testing bacterial resistance was BioMérieux VITEK2, and the results were determined in accordance with the 2017 Clinical and Laboratory Standards Association (CLSI) guidelines (Clinical and Laboratory Standards Institute [CLSI], 2017).

### Comparison of the Single Nucleotide Polymorphisms of the Backbone Sequence and Insertion Site of the Variable Region

The evolution tree of the nucleotide sequence for the single nucleotide polymorphisms (SNPs) of the backbone was inferred using the Maximum Likelihood method. The analyses were conducted using MEGA 7.0.14 software. The insertion gene elements of the prophage variable region were identified using MeV4.9.0 software.

### Sequence Coverage and Identity of Prophage DNA, and Drug Resistance Genes or Mobile Gene Elements of the Variable Region

The hotmaps of sequence identity and coverage of prophage DNA and the comparative study of the drug resistance genes or mobile gene elements in the variable region were drawn with MeV 4.9.0 software.

### Nucleotide Sequence Accession Numbers

Nucleotide sequence accession numbers of the prophage DNA sequences in the four strains, namely, 19051, 675920, 721005, and 911021, and the 16 genomes of *K. pneumoniae* from GenBank are shown in Table 2.

## Results

### Antimicrobial Susceptibility Tests and Transferrable Features

The 16 S rRNA sequences were determined to be *K. pneumoniae* using BLAST. The results of the antimicrobial susceptibility tests on four isolates of *K. pneumoniae* (19051, 675920, 721005, and 911021) are shown in Table 1. After these tests, we used *E. coli* J53 as a recipient strain to conduct conjugative transfer experiments on Φ19051-sap, Φ675920-sap, Φ721005-sap, and Φ911021-sap of *K. pneumoniae* harboring prophage. However, we could not create conjugons carrying prophage despite repeated trials.

**TABLE 1:** Antimicrobial drug susceptibility profiles.

**Category**	**Antimicrobial drug**	**MIC (mg/L)/antimicrobial susceptibility**			
		
		**19051**	**675920**	**721005**	**911021**
Penicillins	Ampicillin	R	R	R	R
	Ampicillin/sulbactam	R	R	R	R
	Piperacillin	R	R	R	R
	Piperacillin/tazobactam	R	R	R	R
Cephalosporins	Cefazolin	R	R	R	R
	Cefuroxime	R	R	R	R
	Cefuroxime axetil	R	R	R	R
	Ceftazidime	R	R	R	R
	Ceftriaxone	R	R	R	R
	Cefepime	R	R	R	R
Monobactam	Aztreonam	R	R	R	R
Carbapenems	Imipenem	R	R	R	R
	Meropenem	R	R	R	R
Aminoglycosides	Amikacin	R	R	S	R
	Gentamicin	R	R	I	R
	Tobramycin	R	R	R	R
Fluoroquinolones	Ciprofloxacin	R	R	R	R
	Levofloxacin	R	R	R	R
Furan	Nitrofurantoin	R	R	I	R
Sulfanilamides	Trimethoprim/Sulfamethoxazole	R	R	R	R

*S = sensitive; R = resistant; I = intermediately resistant.*

### Comparison and Analysis of Prophage DNA

**FIGURE 1:**
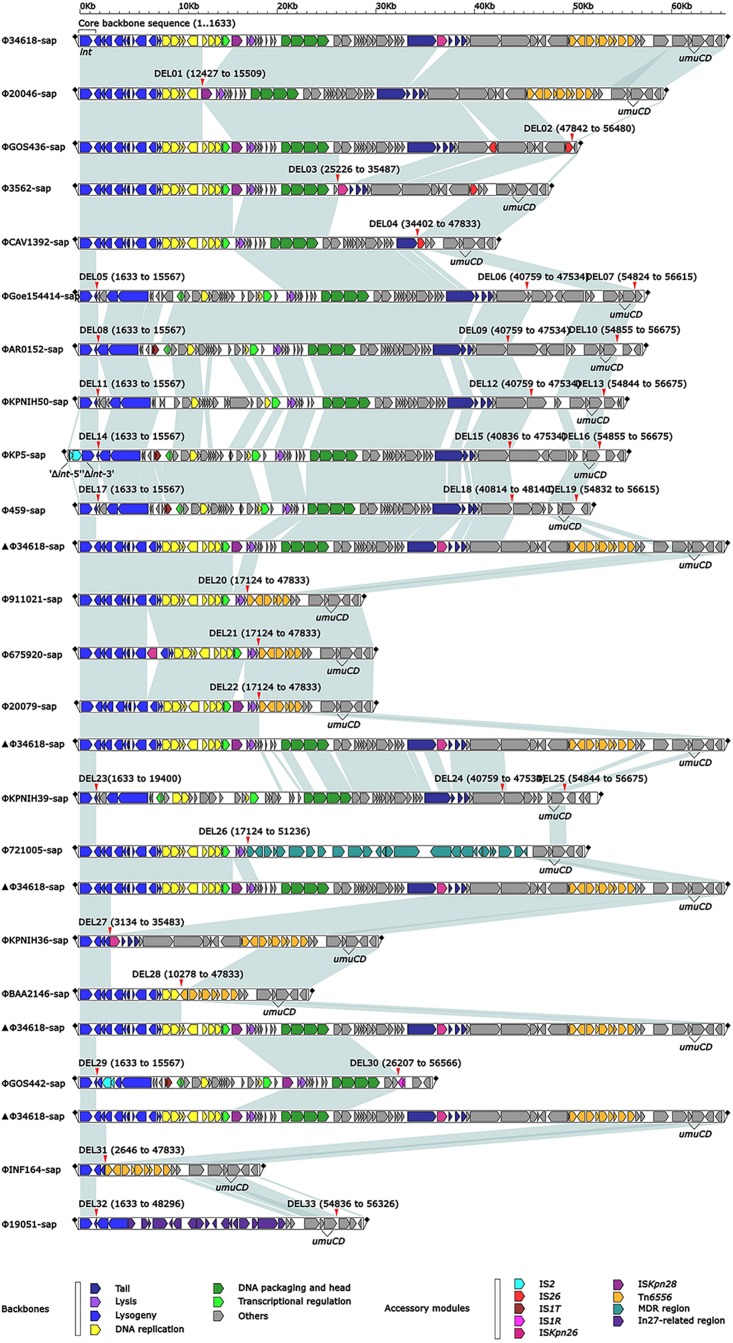
Comparison of linear analysis of the prophage DNA sequences. The linear analysis of the prophage DNA sequences for Φ19051-sap, Φ675920-sap, Φ721005-sap, and Φ911021-sap compared with 16 GenBank prophage DNA sequences (GenBank accession numbers are shown in Table 2). Genes are indicated by arrows; characteristics of function and classification are marked with colors for genes and mobile gene elements. Shaded parts indicate homologous regions (nucleotide homology >95%).

The 20 prophages in this study were all identified from the *K. pneumoniae* chromosomes. A naming convention of ΦXXX-sap was used to indicate where the prophage sequences were inserted into the *sap* sites of the chromosomes. The shortest prophage sequence was 18 kb and the longest was 65 kb, including 39–109 bp open reading frames. The average G + C content in the prophages was 49.6–53.6% (Figure 1, Table 2, and Supplementary Table S1). The region from 1 to 1,633 bp at the 5′ end was the common core backbone region of all sequences. All sequences contained the core genes *int* and *umuCD* except for ΦGOS436-sap and ΦGOS442-sap (Figure 1). The same 16 bp in the forward direction sequences (DRs: target site duplication signals) was confirmed at both sides of all sequences (Figure 1). A more detailed genomic comparison revealed that the same or different sites in the backbones of the prophages were interrupted by various insertion sequences, including IS*1R*, IS*1T*, IS*2*, IS*26*, IS*Kpn26*, IS*Kpn28*, Tn*6556*, MDR, and the In27-related region, except for in ΦGoe154414-sap, ΦKPNIH50-sap, and ΦKPNIH39-sap (Table 2). Compared with Φ34618-sap, the sequences of the backbone regions of the other prophages were gradually lost. Only *int* and *umuCD* were highly conserved core genes (Figure 1).

**TABLE 2:** Major features of prophages analyzed.

**Prophage**	**Category**					
	
	**Accession number**	**Total Length (bp)**	**Total number of ORFs**	**Mean G + C content,%**	**Host bacterium**	**Accessory modules**
Φ34618-sap	CP010392	65,478	109	51.9	*K. pneumoniae*	IS*Kpn28*, IS*Kpn26*, Tn*6556*^#^
Φ20046-sap	CP028783	59,286	94	51.5	*K. pneumoniae*	IS*Kpn28*, Tn*6556*#
ΦGOS436-sap	CP023907	50,549	76	51.2	*K.pneumoniae*	IS*Kpn28*, IS26, IS26
Φ3562-sap	CP025005	47,619	72	51.0	*K.pneumoniae*	IS*Kpn28*, IS*Kpn26*, IS26
ΦCAV1392-sap	CP011578	42,258	62	50.9	*K. pneumoniae*	IS26
ΦGeo154414-sap	CP018337	57,375	73	51.6	*K. pneumoniae*	–
ΦAR0152-sap	CP021944	57,211	73	51.6	*K. pneumoniae*	IS*1T*
ΦKP5-sap	CP012426	56,637	79	51.4	*K. pneumoniae*	IS*2*, IS*1T*
ΦKPNIH50-sap	CP026177	55,270	69	51.9	*K. pneumoniae*	–
Φ459-sap	CP018306	51,879	70	51.3	*K. pneumoniae*	IS*1T*
ΦKPNIH39-sap	CP014762	52,648	63	50.1	*K. pneumoniae*	–
Φ911021-sap	CP022882	28,584	53	51.1	*K. pneumoniae*	ΔTn*6556*#
Φ675920-sap	CP033242	29,785	57	51.2	*K. pneumoniae*	IS*Kpn26*, ΔTn*6556*#
Φ20079-sap	CP029384	29,685	59	51.0	*K. pneumoniae*	IS*Kpn28*, ΔTn*6556*#
ΦKPNIH36-sap	CP014647	30,388	54	52.7	*K. pneumoniae*	IS*Kpn26*, Tn*6556*#
Φ721005-sap	CP022997	51,335	87	49.6	*K. pneumoniae*	MDR region#
ΦGOS442-sap	CP023925	35,881	65	50.5	*K. pneumoniae*	IS*2*, IS*1T*, IS*Kpn28*, IS*1R*
ΦBAA2146-sap	CP006659	23,317	46	51.9	*K. pneumoniae*	ΔTn*6556*#
ΦINF164-sap	CP006659	18,377	46	53.6	*K. pneumoniae*	Tn*6556*#
Φ19051-sap	CP024556	28,884	39	52.1	*K. pneumoniae*	In27-related region#

*^#^Accessory modules containing resistance genes. Detailed data are listed in Supplementary Material.*

### Massive Genetic Acquisitions or Losses Resulting in Evolution of Prophage DNA

**FIGURE 2:**
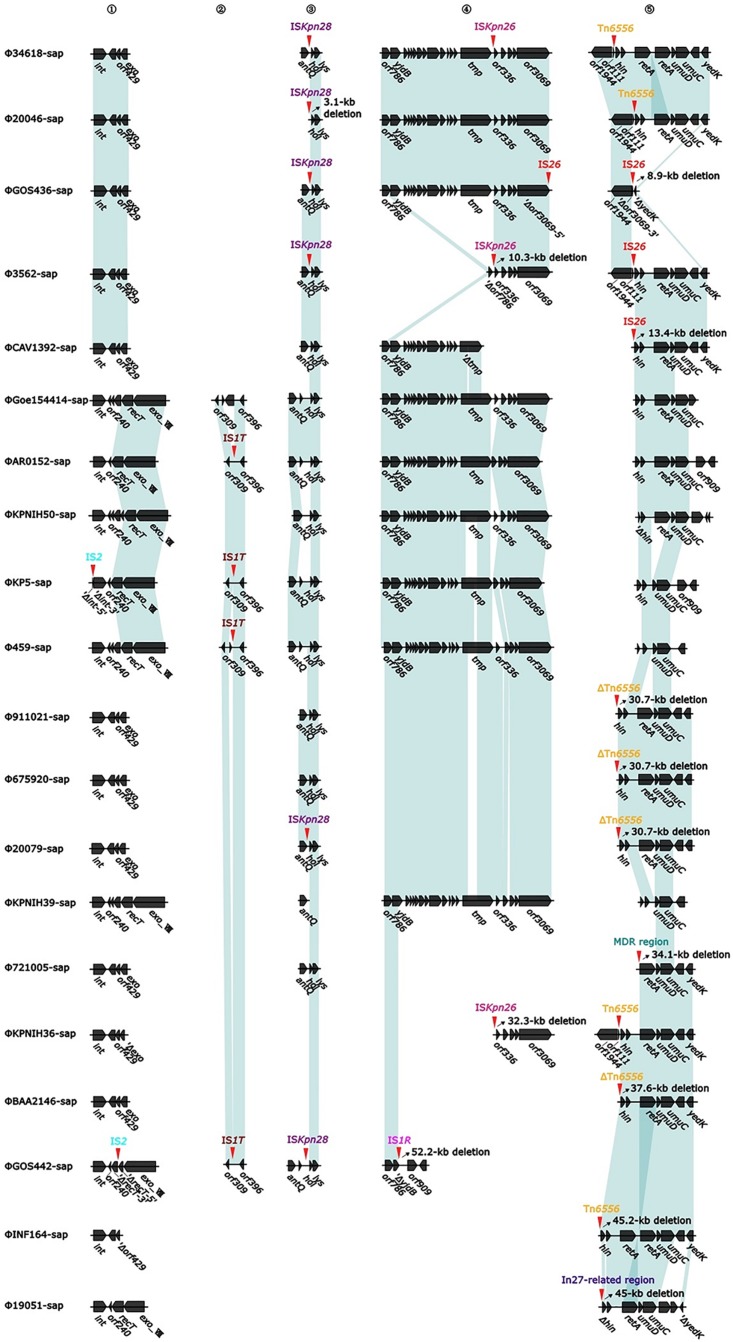
Diagram of the insertion site for accessory modules (variable regions). The genes are indicated by arrows; the shaded parts indicate homologous regions (nucleotide homology >95%). Numbers 1 to 5 represent the hotspots with insertions of variable regions, the red arrow and the mark directly above it indicate the specific position and element of the insertion, and the oblique upward arrow marked on the right side of the part of the red arrow indicates the base of the backbone in the prophage caused by the insertion of the variable region, resulting in deletions of the base (accurate to 0.1 kb).

At least nine large genetic structures that appeared to be from acquisitions or losses were identified in the 20 prophages in this study (Figure 2). First, IS*2* was inserted into the genes *int* (integrase gene) (ΦKP5-sap) and *recT* (recombinant repairing protein RecT) (ΦGOS442-sap), leading to both genes being disrupted into two parts, Δ*int-5*′ and Δ*int-3*′, and Δ*recT-5*′ and Δ*recT-3*′, but no base loss was induced. Second, the insertion of IS*1T* into the *orf309* and *orf396* genes was generally determined; however, these simple insertions might not delete bases in ΦAR0152-sap, ΦKP5-sap, Φ459-sap, and ΦGOS442-sap. Third, IS*Kpn28* was inserted upstream of the *hol* (*holin*) gene, resulting in a 3.1-kb loss in Φ20046-sap, but no base variation occurred in Φ34618-sap, ΦGOS436-sap, Φ3562-sap, Φ20079-sap, and ΦGOS442-sap. Fourth, the insertion of IS*1R* downstream of the *yjdB* gene resulted in a 52.2-kb loss in ΦGOS442-sap. Fifth, the insertion of IS*Kpn26* upstream of the *orf336* gene caused a 10.3-kb loss in Φ3562-sap and a 32.3-kb loss in ΦKPNIH36-sap. Sixth, insertion of Tn*6556* upstream of the *hin* gene resulted in a 35.2-kb loss in each of Φ911021-sap, Φ675920-sap, and Φ20079-sap, a 37.6-kb loss in ΦBAA2146-sap, and a 45.2-kb loss in ΦINF164-sap; however, the insertion of Tn*6556* upstream of the *hin* gene in Φ34618-sap, Φ20046-sap, and ΦKPNIH36-sap did not cause any sequence deletion. Seventh, two IS*26*s were inserted into ΦGOS436-sap, located in the middle and downstream of the *orf3069* gene, splitting this gene into two parts, Δ*orf3069-5*′ and Δ*orf3069-3*′, resulting in an 8.9-kb deletion in ΦGOS436-sap. IS*26* in ΦCAV1392-sap inserted upstream of the *hin* gene resulted in a 13.4-kb loss, while the insertion of IS*26* upstream of the *hin* gene in Φ3562-sap also did not cause any base loss. Eighth, among the 20 sequences analyzed, only Φ721005-sap containing the MDR region, located upstream of the *retA* gene (reverse transcriptase gene), resulted in a 34.1-kb loss, including *hin* and its upstream genes. Ninth, the exogenous insertion into Φ19051-sap of the In27-related region, located between the *hin* and *exoVIII* genes (deoxyribonuclease *exoVIII*), resulted in a 45-kb loss, involving part of the *hin* and *exoVIII* genes.

### The MDR Region From Φ721005-Sap

**FIGURE 3:**
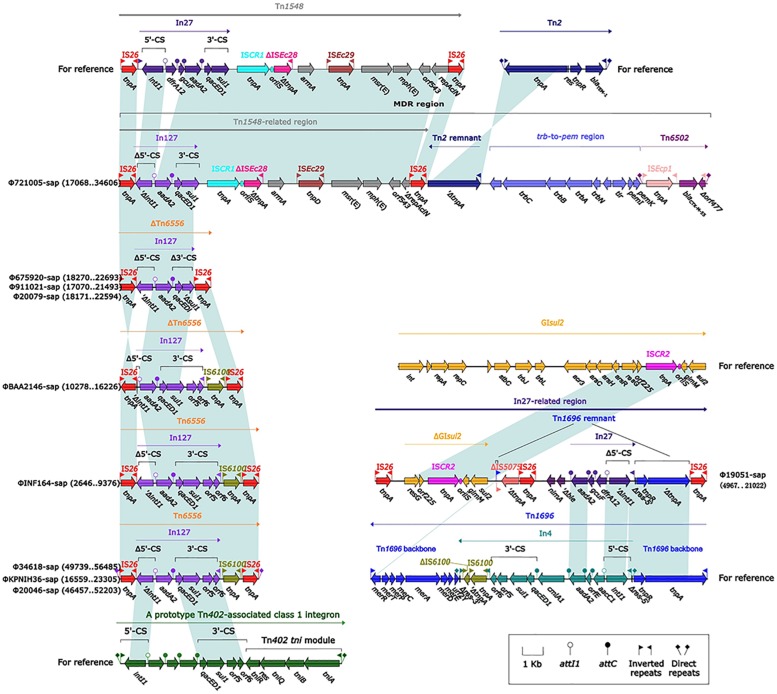
Comparison of an exogenous insertion containing a drug resistance gene and the relevant gene elements. Genes are indicated by arrows; genes, mobile elements, and other features are colored based on function and classification. Shaded regions indicate homologous nucleotides (nucleotide homology >95%); the numbers in parentheses indicate the relative nucleotide site. The exogenous insertion (Tn*6556*) with the identified sequence is included in the Figure at the lower left part.

The largest number of drug resistance genes in Φ721005-sap was found to be located in the MDR region (Figure 3), followed by the Tn*1548*-related region, in which In27 in the original Tn*1548* was replaced by In127, the truncated *repAciN* gene, and a 7.2-kb-long unknown functional region *trb*-to-*pem*, a 2.5-kb-long Tn*2* remnant (including Tn*2* IRL and truncated *tnpA*), and complete Tn*6502* (containing the beta-lactamase resistance gene *bla*_*CTX–M–*__55_). Tn*1548* was shown to be a composite transposon flanked by IS*26* with no forward direction repeats (DRs) at both ends and its structure was IS*26*-In27-IS*CR1*-ΔIS*Ec28*-*armA* (aminoglycoside resistance gene)-IS*Ec29*-*msr(E)*- *mph(E)* (macrolide resistance gene)-*orf543*-*repAciN*-IS*26*. In27 in Tn*1548* could be replaced by different class 1 integrons, forming many Tn*1548*-related elements (González-Zorn et al., 2005; Du et al., 2012). In127, unlike In27, had only one *aadA2* (aminoglycoside resistance gene) gene cassette (Figure 3).

### Evolution of the In27-Related Region From Φ19051-Sap

Tn*1696* belongs to the Tn*21* subgroup of the Tn*3* transposon family. It was produced by insertion of the dissociation site (*res*) into the core backbone region by class I integron In4, and its structure was shown to be IRL (left reverse repeat)-*tnpA* (transposase)-*tnpR* (dissociation enzyme)-*res* (dissociation site)-*mer* (mercury resistance site)-IRR (right reverse repeat sequence) (Partridge et al., 2001). The In27-related region in Φ19051-sap was derived from Tn*1696*, which was produced by five major evolutionary events in Tn*1696* as follows. First, the truncated IS*5075* was inserted into the middle of the IRR, resulting in only a 22-bp remnant in the IRR. Second, the incomplete In27 was inserted into the same *res* site of In4, and the *res* was truncated to the 5′ end as a 75-bp remnant. The sequences of the In27 and In4 gene cassettes were completely different; the In27 gene cassette featured *aadA2*-*gcuF*-*dfrA12*, while the In4 gene cassette contained *cmlA1*-*aadA2*-*orfE*-*aacC1*. Moreover, the 3′-CS of In27 was absent. *AadA2* and *dfrA1* are genes conferring aminoglycoside resistance and trimethoprim resistance, respectively, and *gcuF* encodes a pseudo protein. The other three events included the insertion of Δ*ble* (bleomycin resistance gene) and *nimA* (unknown function), the deletion of the *mer* gene, and the replacement of IS*26* and ΔIS*5075* by IS*6100*. Downstream of IS*5075* was ΔGI*sul2* (3′ end of GI*sul2*: *resG*-*orf225*-IS*CR2*-*glmM*-*sul2*) and IS*26*. GI*sul2* is a large mobile element carrying integrase (*int*), including the resolvase gene *resG*, several conjugative transfer genes, sulfonamide resistance gene *sul2*, and IS*CR2* sequences, and is present in various bacteria (Nigro and Hall, 2011).

### The Novel Composite Transposon Tn*6556*

Tn*6556* is a novel composite transposon first discovered in Φ34618-sap, ΦKPNIH36-sap, and Φ20046-sap (Figure 3). Tn*6556* consists of class I integron In127 and three independent inserts, IS*26*-In127 (Δ*intl1*-*aadA2*-*qacED1*-*sul1*-*orf5*-*orf6*)- IS*6100*-IS*26*, and 8-bp forward DRs on both ends. Further truncation of the Δ*intl1* gene made Tn*6556* incomplete in ΦBAA2146-sap. The deletion of IS*6100* and its upstream *orf6* and *orf5*, together with the truncation of *sul1*, resulted in significant truncation of Tn*6556* in Φ675920-sap, Φ911021-sap, and Φ20079-sap, but the *aadA2* gene remained intact. No forward DRs were found on both ends of Tn*6556* in ΦINF164-sap, ΦBAA2146-sap, Φ675920-sap, Φ911021-sap, and Φ20079-sap. The classical structure of the prototype for the Tn*402*-associated class I integron is IRi (integron end inverted direction repeat) -5′-CS [5′-conserved fragment: *intI1* (integrase), *attI1* (specific recombination site), GC (gene cassette) array], 3′-CS [3′-conserved fragment: *qacED1*-*sul1*-*orf5*-*orf6*], a Tn*402tni* module [*tniA* (transposase) -*tniB* (ATP-binding protein) -*tniQ* (transposition auxiliary protein) -*res*-*tniR* (serine dissociation enzyme)], and IRt (*tni* terminal inverted direction repeat). In127 contained a truncated 5′-CS, a gene cassette, and a complete 3′-CS, but did not involve a Tn*402tni* module. The transposases of IS*26* and IS*6100* on both sides of Tn*6556* might mediate transposon mobility (Roberts et al., 2008).

### Evolutionary Analysis of the Prophage DNA Sequences

**FIGURE 4:**
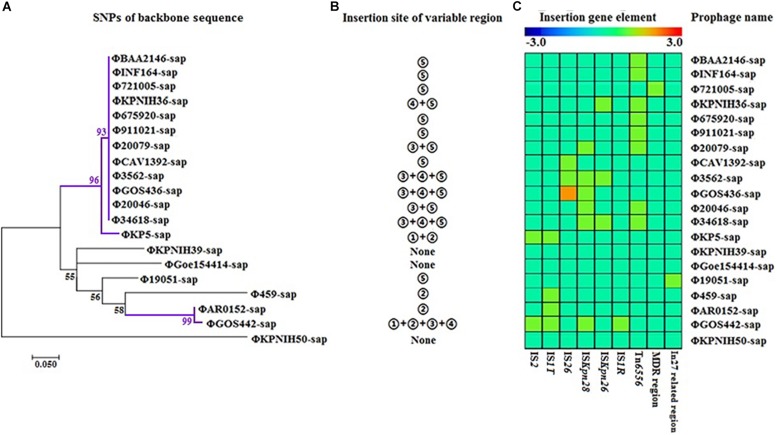
Evolutionary relationships of prophage DNA for four strains of *K. pneumoniae* DNA and 16 prophage DNAs identified from GenBank. **(A)** Evolution tree of prophage backbone. The evolutionary history was inferred using the Maximum Likelihood method. **(B)** Insertion site of the variable region. Insertion sites ➀, ➁,➂, ➃, and ➄ are identical to those of Figure 2. **(C)** Gene elements of the prophage variable region. Data is also collected from Figure 2.

**FIGURE 5:**
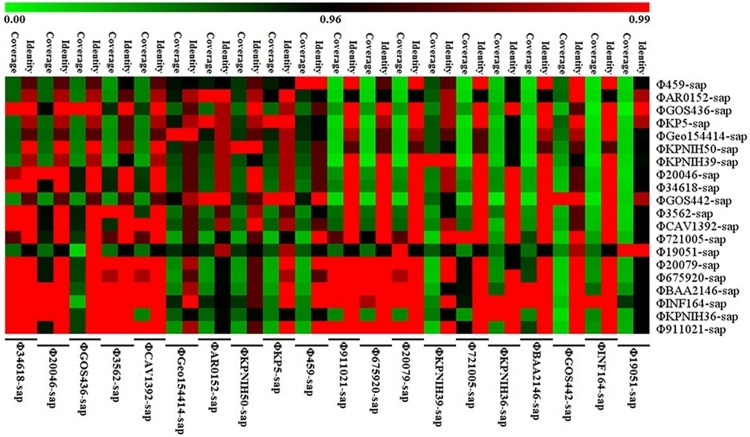
Sequence coverage and identity among the prophage DNAs for four strains of *K. pneumoniae* and 16 prophage DNAs identified from GenBank. There is high sequence identity (average nucleotide homology >95%), but various coverage among the 20 prophage DNAs based on Supplementary Table S1.

**FIGURE 6:**
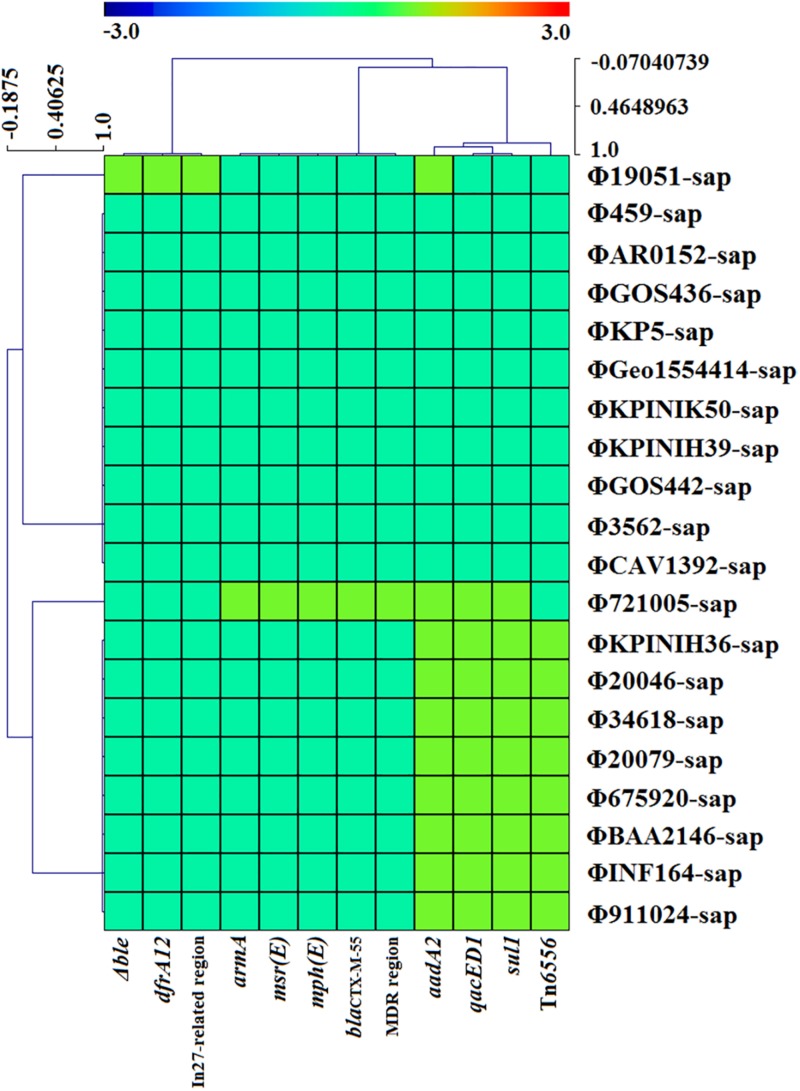
The correlativity of drug resistance genes or mobile gene elements are determined with *CARD*, *ResFinde*r, and Tn Number Registry databases among the four strains of *K. pneumoniae* and 16 prophage DNAs identified from GenBank. There are only 10 prophage DNAs harboring drug resistance genes, which are generally located on mobile gene elements such as the MDR region, In27-related region and novel Tn*6556*.

The evolutionary relationships of the 20 prophage DNAs are shown in Figures 4A–C, 5, 6. The identities of the related SNPs found in the backbone sequence were obtained with high confidence among the ΦBAA2146-sap, ΦINF164-sap, Φ721005-sap, Φ675920-sap, Φ911021-sap, Φ20079-sap, ΦCAV1392-sap, Φ3562-sap, ΦGOS436-sap, Φ20046-sap, and Φ34618-sap (Figure 4A, all identified above a 93% confidence level, purple line), and the ΦKP5-sap (Figure 4A, identified at a 96% confidence level, purple line). The identities of the related SNPs found in ΦAR0152-sap and ΦGOS442-sap were obtained at a 99% confidence level (Figure 4A, purple line). These results suggested that this region of the backbone sequence was largely homologous. However, regions of the backbone sequence where the identities of the SNPs were associated with a confidence level of less than 60% indicated that the nucleotide sequence of that backbone region was diverse compared with the above region. These results are consistent with the elements and sites of insertion shown in Figures 4B,C. Figure 4B shows the insertion sites of the genes or gene elements. Figure 4C graphically shows the inserted genes or gene elements. Taken together, Figure 4 summarizes the evolution and changes of the 20 prophage DNAs, including the sites and types of inserted genes or gene elements, suggesting there are contradictory features in phage DNA stability and variation.

Figure 5 illustrates the high identity of the partial sequence in the 20 prophage DNAs, indicating an average nucleotide identity (homology) of >95%, consistent with the shaded regions in Figures 1–3. However, the various coverages of the 20 prophage DNAs vividly suggest the polymorphism of evolution and change. Figure 6 reveals the relationship among the drug resistance genes or gene elements in the variable region, suggesting the ever-changing characteristics of gene elements. These results suggest the high sequence identification and homology of prophage DNA and helped to characterize the evolutionary diversity of the prophage DNA examined in this study.

## Discussion

The analysis of four prophage DNA sequences and 16 similar prophage DNA sequences from GenBank indicated that they were all specifically inserted into the *sap* sites of *K. pneumoniae* chromosomes. It is well known that *sapABC* is a permease of the peptide transport system, which mediates the transport of phages or particles into *K. pneumoniae* cells; accordingly, the capsule biosynthesis of phage in *K. pneumoniae* is productive and regulated (Dorman et al., 2018). The *sap ABC* transporter promotes capsule production by increasing gene expression in the middle of the capsule (Dorman et al., 2018). Then, the *sapABC* system replicates and inserts phage DNA into the *K. pneumoniae* DNA, forming prophage DNA. Over time, after experiencing the above-mentioned massive genetic acquisitions and losses, including changes to the lysogenic transformation regions, DNA replication regions, and transcriptional regulatory regions of the prophage, among others, the bases of the backbone regions were gradually lost over the course of evolution. This would increase excessive insertions with or without drug resistance genes or gene elements such as IS*1T*, IS*1R*, IS*26*, IS*Kpn26*, IS*Kpn28*, Tn*6556*, In27-related, and the MDR region. These factors enable the host bacteria to reduce their metabolism and external pressures, promoting better adaptation to the environment, and revealing the evolutionary benefits of prophage DNAs.

Over the course of prophage evolution, the *int* gene has always been present, suggesting that it is the most conserved core of the backbone region. Owing to the evolutionary selective pressures present during the process of integrating DNA into host bacteria, the prophage DNA inevitably undergoes gene mutations and deletions. Transfer of prophage DNA requires two reactions: first, excision of the prophage DNA from the host bacterium and second, integration of the prophage into a new host bacterium. The excision of the prophage involves a functional *xis* gene, but this is not absolute. If there is no Xis enzyme, the integration enzyme (Int) and the integral host factor (IHF) can complete the transfer of the prophage DNA. Integrating a prophage into a new host requires a functional integrase (Int) followed by a specific binding site (*attB*) in the host, and any nucleotide changes in the core region of the binding site might result in failure of the prophage cell integration (Nash, 1981). Likewise, if the conjugate was integrated into a bacterial cell, the phage could express a protein that was toxic to the cell and kill all exconjugants. Therefore, the failure of the conjugate transfer experiment in this study is not surprising. It is generally accepted that DNA excision depends on phage-encoded proteins, and the excision enzyme (Xis) and integrase (Int) are considered to be involved in the first step in prophage induction, allowing the prophage DNA to be excised and then replicated (Davidson, 2018). However, it has also been reported that several prophages of *Staphylococcus aureus* were found to have significantly delayed transcription of the *xis* gene compared with that of the genes encoding the proteins required for DNA replication and prophage-particle production. The result of this delay was the replication of prophage DNA *in situ* within the bacterial genome and subsequent encapsulation of the prophage DNA, which was still attached to the adjacent bacterial DNA (Chen et al., 2018; Davidson, 2018).

Based on the genes or genetic mobile elements investigated, some of prophages appeared functional and were even phenotypically expressed. Comparative analysis of the 20 prophage genomes from the *sap* site of the *K. pneumoniae* chromosome indicated that a positive repeat of 16 bp in length was observed at both ends of all prophages, and the sequences were identical, labeled as *attL* and *attR* (Figure 1, a small square flag at both ends of the sequence). Compared with the reference sequence Φ34618-sap, the sequence of the backbone regions of the other prophages was gradually lost. However, the most stable core genes were *int* and *umuCD* (Figure 1). At least nine exogenous insertion regions including IS*1R*, IS*1T*, IS*2*, IS*26*, IS*Kpn26*, IS*Kpn28*, Tn*6556*, MDR, and In27-related regions were determinate in this study (Table 1), in which Tn*6556*, MDR, and the In27-related regions were involved as resistance genes. Additionally, a novel composite transposon Tn*6556* was discovered in the *K. pneumoniae* chromosome. Different insertion elements of a series of exogenous insertion regions including IS and Tn on the prophage had relatively specific insertion sites where IS*Kpn28* was located upstream of the *hol* gene, IS*Kpn26* was located upstream of the *orf* gene, and Tn*6556* was located upstream of the *hin* gene. As the simplest transposition element, the shearing and transfer of IS was accomplished by its own transposase. Tn*6556* was flanked by two repeating IS*26s*, and this repetitive IS mediated homologous recombination of Tn*6556*, and also resulted in genetic loses near the recombination site resulting in diversity in the prophage DNA.

Tn*6556* containing In127 was discovered for the first time in this study (Tables 2, 3 and Figures 2–4, 6). The results of the antimicrobial susceptibility test shown in Table 1 indicated that the *K. pneumoniae* strains were highly resistant to various antimicrobial agents, suggesting that in addition to the phenotypic expression of the resistance genes or gene elements carried on the chromosome, one or more plasmids carrying resistance genes or gene elements might also be involved (Tables 1–3 and Figures 1–3, 6), and warrants further study. The results of Figure 2 summarizing the five sites of base insertion and nine types of base acquisition or loss in prophage DNA (Table 2 and Figures 2, 3, 4B,C) show in detail the molecular mechanism behind the evolution of the prophage DNA and the changes of AMR (Table 3 and Figure 6). AMR encoded in a bacterial chromosome might originate from new mutations or acquired AMR genes, which could be maintained by replication without selection pressure (Navon-Venezia et al., 2017). This process would facilitate the evolutionary adaptation of bacteria and has a potential impact on the monitoring and treatment of bacterial infections (Shen et al., 2019). Our results confirm that AMR genes are encoded in the chromosome of *K. pneumoniae* (Mathers et al., 2017), and they can be classified as “core AMR gene” or “acquired AMR gene” (Wyres and Holt, 2016). Furthermore, these results indicate that the incorporation of phage DNA has resulted in a diverse set of *K. pneumoniae* chromosomes, confirming their ever-changing roles in the evolutionary success of the bacterium. This study did not examine how the phages of the relevant gene locus are induced and transferred to other strains, and how these phages play a role in the transfer of AMR genes, and therefore these questions require further investigation in future studies.

**TABLE 3:** Drug resistance genes in mobile elements analyzed in this study.

**Prophage**	**Resistance marker**	**Resistance phenotype**	**Nucleotide position**	**Region located**
Φ34618-sap	*aadA2*	Aminoglycoside resistance	51522..52301	Tn*6556*
	*qacED1*	Quaternary ammoniumcompound resistance	52465..52812	
	*sul1*	Sulfonamide resistance	52806..53645	
Φ20046-sap	*aadA2*	Aminoglycoside resistance	47240..48019	Tn*6556*
	*qacED1*	Quaternary ammoniumcompound resistance	48183..48530	
	*sul1*	Sulfonamide resistance	48524..49363	
Φ911021-sap	*aadA2*	Aminoglycoside resistance	18831..19610	ΔTn*6556*
	*qacED1*	Quaternary ammoniumcompound resistance	19774..20121	
	Δ*sul1*	Sulfonamide resistance	20115..20673	
Φ675920-sap	*aadA2*	Aminoglycoside resistance	20032..20811	ΔTn*6556*
	*qacED1*	Quaternary ammoniumcompound resistance	20975..21322	
	Δ*sul1*	Sulfonamide resistance	21316..21874	
Φ20079-sap	*aadA2*	Aminoglycoside resistance	19932..20711	ΔTn*6556*
	*qacED1*	Quaternary ammoniumcompound resistance	20875..21222	
	Δ*sul1*	Sulfonamide resistance	21216..21774	
ΦKPNIH36-sap	*aadA2*	Aminoglycoside resistance	18342..19121	Tn*6556*
	*qacED1*	Quaternary ammoniumcompound resistance	19285..19632	
	*sul1*	Sulfonamide resistance	19626..20465	
Φ721005-sap	*aadA2*	Aminoglycoside resistance	18830..19609	MDR region
	*qacED1*	Quaternary ammoniumcompound resistance	19773..20120	
	*sul1*	Sulfonamide resistance	20114..20953	
	*armA*	Aminoglycoside resistance	24295..25068	
	*msr(E)*	Macrolide resistance	27367..28842	
	*mph(E)*	Macrolide resistance	28898..29782	
	*bla*_*CTX–M–*__55_	β-lactam resistance	44246..45121	
ΦBAA2146-sap	*aadA2*	Aminoglycoside resistance	11271..12050	ΔTn*6556*
	*qacED1*	Quaternary ammoniumcompound resistance	12214..12561	
	*sul1*	Sulfonamide resistance	12555..13394	
ΦINF164-sap	*aadA2*	Aminoglycoside resistance	4421..5200	Tn*6556*
	*qacED1*	Quaternary ammoniumcompound resistance	5364..5711	
	*sul1*	Sulfonamide resistance	5705..6544	
Φ19051-sap	Δ*ble*	Bleomycin resistance	12881..13272	In27-related region
	*aadA2*	Aminoglycoside resistance	14469..15248	
	*dfrA12*	Trimethoprim resistance	15668..16165	

*Detailed data are listed in Supplementary Material.*

## Conclusion

The emerging diversity of prophages is the result of base acquisition or loss with a large number of variable regions being associated with insertions of different prophage DNA. This has resulted in various degrees of base loss in the backbone region near the insertion sites, conferring evolutionary diversity on prophage DNA.

## Data Availability Statement

The datasets generated for this study can be found in the GenBank.

## Author Contributions

DW and FW conceptualized and designed the study. FW, WH, QJ, JF, and DZ acquired the data. DW, FW, JF, and DZ analyzed and interpreted the data. DW and FW drafted the manuscript. DW and DZ critically revised the manuscript. All authors read and approved the final manuscript.

## Conflict of Interest

The authors declare that the research was conducted in the absence of any commercial or financial relationships that could be construed as a potential conflict of interest.

## Footnotes

1 https://github.com/ruanjue/smartdenovo

2 https://inkscape.org/en

## References

[B1] Alanis A. J. (2005). Resistance to antibiotics: are we in the post-antibiotic era? *Arch. Med. Res.* 36 697–705. 10.1016/j.arcmed.2005.06.009 16216651

[B2] Boratyn G. M. Camacho C. Cooper P. S. Coulouris G. Fong A. Ma N. (2013). BLAST: a more efficient report with usability improvements. *Nucleic Acids Res.* 41 29–33. 10.1093/nar/gkt282 23609542 PMC3692093

[B3] Boutet E. Lieberherr D. Tognolli M. Schneider M. Bansal P. Bridge A. J. (2016). UniProtKB/Swiss-Prot, the manually annotated section of the UniProt KnowledgeBase: how to use the entry view. *Methods Mol. Biol.* 1374 23–54. 10.1007/978-1-4939-3167-5_2 26519399

[B4] Brettin T. Davis J. J. Disz T. Edwards R. A. Gerdes S. Olsen G. J. (2015). RASTtk: a modular and extensible implementation of the RAST algorithm for building custom annotation pipelines and annotating batches of genomes. *Sci. Rep.* 5:8365. 10.1038/srep08365 25666585 PMC4322359

[B5] Brown-Jaque M. Calero-Cáceres W. Muniesa M. (2015). Transfer of antibiotic- resistance genes via phage-related mobile elements. *Plasmid* 79 1–7. 10.1016/j.plasmid.2015.01.001 25597519

[B6] Brüssow H. Canchaya C. Hardt W. D. (2004). Phages and the evolution of bacterial pathogens: from genomic rearrangements to lysogenic conversion. *Microbiol. Mol. Biol. Rev.* 68 560–602. 10.1128/MMBR.68.3.560-602.2004 15353570 PMC515249

[B7] Bushman F. (2002). *Lateral DNA Transfer: Mechanisms and Consequences.* Cold Spring Harbor, NY: Cold Spring Harbor Laboratory Press.

[B8] Calero-Cáceres W. Ye M. Balcázar J. L. (2019). Bacteriophages as environmental reservoirs of antibiotic resistance. *Trends Microbiol.* 27 570–577. 10.1016/j.tim.2019.02.008 30905524

[B9] Castillo D. Kauffman K. Hussain F. Kalatzis P. Rørbo N. Polz M. F. (2018). Widespread distribution of prophage-encoded virulence factors in marine Vibrio communities. *Sci. Rep.* 8:9973. 10.1038/s41598-018-28326-9 29967440 PMC6028584

[B10] Chen J. Quiles-Puchalt N. Chiang Y. N. Bacigalupe R. Fillol-Salom A. Chee M. S. J. (2018). Genome hypermobility by lateral transduction. *Science* 362 207–212. 10.1126/science.aat5867 30309949

[B11] Clinical and Laboratory Standards Institute [CLSI] (2017). *Performance Standards for Antimicrobial Susceptibility Testing: twenty-seventh informational supplement M100-S27.* Wayne, PA: CLSI.

[B12] Davidson A. R. (2018). A common trick for transferring bacterial DNA. *Science* 362 152–153. 10.1126/science.aav1723 30309931

[B13] Davies E. V. Winstanley C. Fothergill J. L. James C. E. (2016). The role of temperate bacteriophages in bacterial infection. *FEMS Microbiol. Lett.* 363:fnw015. 10.1093/femsle/fnw015 26825679

[B14] Dcosta V. M. King C. E. Kalan L. Morar M. Sung W. W. L. Schwarz C. (2011). Antibiotic resistance is ancient. *Nature* 477 457–461. 10.1038/nature10388 21881561

[B15] Dedrick R. M. Guerrero-Bustamante C. A. Garlena R. A. Russell D. A. Ford K. Harris K. (2019). Engineered bacteriophages for treatment of a patient with a disseminated drug-resistant *Mycobacterium abscessus*. *Nat. Med.* 25 730–733. 10.1038/s41591-019-0437-z 31068712 PMC6557439

[B16] Deutsch D. R. Utter B. Verratti K. J. Sichtig H. Tallon L. J. Fischetti V. A. (2018). Extra-chromosomal DNA sequencing reveals episomal prophages capable of impacting virulence factor expression in *Staphylococcus aureus*. *Front. Microbiol.* 9:1406. 10.3389/fmicb.2018.01406 30013526 PMC6036120

[B17] Dorman M. J. Feltwell T. Goulding D. A. Parkhill J. Short F. L. (2018). The capsule regulatory network of *Klebsiella pneumoniae* defined by density- TraDISort. *mBio* 9:e01863-18. 10.1128/mBio.01863-18 30459193 PMC6247091

[B18] Du X. D. Li D. X. Hu G. Z. Wang Y. Shang Y. H. Wu C. M. (2012). Tn*1548*-associated *armA* is co-located with *qnrB2*, *aac(6′)-Ib-cr* and *bla*CTX-M-3 on an IncFII plasmid in a *Salmonella enterica* subsp. enterica serovar Paratyphi B strain isolated from chickens in China. *J. Antimicrob. Chemother.* 67 246–248. 10.1093/jac/dkr407 21965429

[B19] Edgar R. C. (2004). MUSCLE: multiple sequence alignment with high accuracy and high throughput. *Nucleic Acids Res.* 32 1792–1797. 10.1093/nar/gkh340 15034147 PMC390337

[B20] Feng W. Zhou D. Wang Q. Luo W. Zhang D. Sun Q. (2016). Dissemination of IMP-4-encoding pIMP-HZ1-related plasmids among *Klebsiella pneumoniae* and *Pseudomonas aeruginosa* in a Chinese teaching hospital. *Sci. Rep.* 6:33419. 10.1038/srep33419 27641711 PMC5027574

[B21] Frank J. A. Reich C. I. Sharma S. Weisbaumm J. S. Wilson B. A. Olsen G. J. (2008). Critical evaluation of two primers commonly used for amplification of bacterial 16 S rRNA genes. *Appl. Environ. Microbiol.* 74 2461–2470. 10.1128/aem.02272-07 18296538 PMC2293150

[B22] Furfaro L. L. Payne M. S. Chang B. J. (2018). Bacteriophage therapy: clinical trials and regulatory hurdles. *Front. Cell Infect. Microbiol.* 8:376. 10.3389/fcimb.2018.00376 30406049 PMC6205996

[B23] González-Zorn B. Teshager T. Casas M. Porrero M. C. Moreno M. A. Courvalin P. (2005). ArmA and aminoglycoside resistance in *Escherichia coli*. *Emerg. Infect. Dis.* 11 954–956. 10.3201/eid1106.040553 15963296 PMC3367600

[B24] Hackl T. Hedrich R. Schultz J. Förster F. (2014). Proovread: large-scale high- accuracy PacBio correction through iterative short read consensus. *Bioinformatics* 30 3004–3011. 10.1093/bioinformatics/btu392 25015988 PMC4609002

[B25] Holt K. E. Wertheim H. Zadoks R. N. Baker S. Whitehouse C. A. Dance D. (2015). Genomic analysis of diversity, population structure, virulence, and antimicrobial resistance in *Klebsiella pneumoniae*, an urgent threat to public health. *PNAS* 112 E3574–E3581. 10.1073/pnas.1501049112 26100894 PMC4500264

[B26] Keen E. C. Dantas G. (2018). Close encounters of three kinds: bacteriophages, commensal bacteria, and host immunity. *Trends Microbiol.* 26 943–954. 10.1016/j.tim.2018.05.009 29909042 PMC6436384

[B27] Kortright K. E. Chan B. K. Koff J. L. Turner P. E. (2019). Phage therapy: a renewed approach to combat antibiotic-resistant bacteria. *Cell Host Microbe* 25 219–232. 10.1016/j.chom.2019.01.014 30763536

[B28] Lewis K. (2013). Platforms for antibiotic discovery. *Nat. Rev. Drug. Discov.* 12 371–387. 10.1038/nrd3975 23629505

[B29] Liang Q. Yin Z. Zhao Y. Liang L. Feng J. Zhan Z. (2017). Sequencing and comparative genomics analysis of the IncHI2 plasmids pT5282-*mphA* and p112298-*catA* and the IncHI5 plasmid pYNKP001-*dfrA*. *Int. J. Antimicrob. Agent.* 49 709–718. 10.1016/j.ijantimicag.2017.01.021 28390961

[B30] Mathers A. J. Stoesser N. Chai W. Carroll J. Barry K. Cherunvanky A. (2017). Chromosomal integration of the *Klebsiella pneumoniae* carbapenemase gene, *bla*KPC, in *Klebsiella* species is elusive but not rare. *Antimicrob. Agents Chemother.* 61:e01823-16. 10.1128/aac.01823-16 28031204 PMC5328509

[B31] Moura A. Soares M. Pereira C. Leitão N. Henriques I. Correia A. (2009). INTEGRALL: a database and search engine for integrons, integrases and gene cassettes. *Bioinformatics* 25 1096–1098. 10.1093/bioinformatics/btp105 19228805

[B32] Nash H. A. (1981). Integration and excision of bacteriophage λ: the mechanism of conservative site specific recombination. *Annu. Rev. Genet.* 15 143–167. 10.1146/annurev.ge.15.120181.001043 6461289

[B33] Navon-Venezia S. Kondratyeva K. Carattoli A. (2017). *Klebsiella pneumoniae*: a major worldwide source and shuttle for antibiotic resistance. *FEMS. Microbiol. Rev.* 41 252–275. 10.1093/femsre/fux013 28521338

[B34] Nederbragt A. J. (2014). On the middle ground between open source and commercial software - the case of the Newbler program. *Genome Biol.* 15:113. 10.1186/gb4173 25180324 PMC4054848

[B35] Nesme J. Cecillon S. Delmont T. O. Monier J. M. Vogel T. M. Simonet P. (2014). Large-scale metagenomic-based study of antibiotic resistance in the environment. *Curr. Biol.* 24 1096–1100. 10.1016/j.cub.2014.03.036 24814145

[B36] Nigro S. J. Hall R. M. (2011). *GIsul2*, a genomic island carrying the *sul2* sulphonamide resistance gene and the small mobile element CR2 found in the *Enterobacter cloacae* subspecies cloacae type strain ATCC 13047 from 1890, *Shigella flexneri* ATCC 700930 from 1954 and *Acinetobacter baumannii* ATCC 17978 from 1951. *J. Antimicrob. Chemother.* 66 2175–2176. 10.1093/jac/dkr230 21653606

[B37] O’Leary N. A. Wright M. W. Brister J. R. Ciufo S. Haddad D. McVeigh R. (2016). Reference sequence (RefSeq) database at NCBI: current status, taxonomic expansion, and functional annotation. *Nucleic Acids Res.* 44 733–745. 10.1093/nar/gkv1189 26553804 PMC4702849

[B38] Partridge S. R. Brown H. J. Stokes H. W. Hall R. M. (2001). Transposons Tn1696 and Tn21 and their integrons In4 and In2 have independent origins. *Antimicrob. Agents. Chemother.* 45 1263–1270. 10.1128/aac.45.4.1263-1270.2001 11257044 PMC90453

[B39] Pendleton J. N. Gorman S. P. Gilmore B. F. (2013). Clinical relevance of the ESKAPE pathogens. *Expert. Rev. Anti. Infect. Ther.* 11 297–308. 10.1586/eri.13.12 23458769

[B40] Rice L. B. (2010). Progress and challenges in implementing the research on ESKAPE pathogens. *Infect. Cont. Hosp. Epidemiol.* 31 S7–S10. 10.1086/655995 20929376

[B41] Roberts A. P. Chandler M. Courvalin P. Guédon G. Mullany P. Pembroke T. (2008). Revised nomenclature for transposable genetic elements. *Plasmid* 60 167–173. 10.1016/j.plasmid.2008.08.001 18778731 PMC3836210

[B42] Shen J. Zhou J. Xu Y. Xiu Z. (2019). Prophages contribute to genome plasticity of *Klebsiella pneumoniae* and may involve the chromosomal integration of ARGs in CG258. *Genomics* 10.1016/j.ygeno.2019.06.016 [Epub ahead of print]. 31220585

[B43] Siguier P. Perochon J. Lestrade L. Mahillon J. Chandler M. (2006). ISfinder: the reference centre for bacterial insertion sequences. *Nucleic Acids Res.* 34 32–36. 10.1093/nar/gkj014 16381877 PMC1347377

[B44] Sweere J. M. Van Belleghem J. D. Ishak H. Bach M. S. Popescu M. Sunkari V. (2019). Bacteriophage trigger antiviral immunity and prevent clearance of bacterial infection. *Science* 363 1–12. 10.1126/science.aat9691 30923196 PMC6656896

[B45] Torres-Barceló C. (2018). The disparate effects of bacteriophages on antibiotic- resistant bacteria. *Emerg. Microbes Infect.* 7:168. 10.1038/s41426-018-0169-z 30302018 PMC6177407

[B46] Wang X. Kim Y. Ma Q. Hong S. H. Pokusaeva K. Sturino J. M. (2010). Cryptic prophages help bacteria cope with adverse environments. *Nat. Commun.* 1:147. 10.1038/ncomms1146 21266997 PMC3105296

[B47] Wyres K. L. Holt K. E. (2016). *Klebsiella pneumoniae* population genomics and antimicrobial-resistant clones. *Trends Microbiol.* 24 944–956. 10.1016/j.tim.2016.09.007 27742466

[B48] Zankari E. Hasman H. Cosentino S. Vestergaard M. Rasmussen S. Lund O. (2012). Identification of acquired antimicrobial resistance genes. *J. Antimicrob. Chemother.* 67 2640–2644. 10.1093/jac/dks261 22782487 PMC3468078

